# Associations between blood systemic inflammatory markers and anxiety in *Helicobacter pylori*-infected patients

**DOI:** 10.3389/fpsyt.2025.1671858

**Published:** 2025-10-23

**Authors:** Jiantao Gong, Fengjuan Chen, Bin Jiang, Bin Zhou, Xiang Ma, Meilei Wang, Qingyong Dai, Shiming Wang, Xiaojun Yang, En Zhao

**Affiliations:** ^1^ Department of Gastroenterology, Xishan People’s Hospital of Wuxi City, Wuxi, China; ^2^ Department of Neurology, Xishan People’s Hospital of Wuxi City, Wuxi, China

**Keywords:** anxiety, glial fibrillary acidic protein, *Helicobacter pylori*, neuroinflammation, systemic inflammatory markers

## Abstract

**Background:**

Chronic Helicobacter pylori (H. pylori) infection is associated with both gastrointestinal symptoms and systemic inflammation, which may contribute to the development of psychiatric disorders, particularly anxiety. Appropriate psychiatric interventions have been shown to significantly enhance treatment outcomes in patients undergoing clinical management for H. pylori infection. Early screening for anxiety in this population is therefore of critical clinical importance. This study aimed to identify potential biomarkers for anxiety detection and evaluate the relationship between these biomarkers and anxiety symptoms in H. pylori-positive individuals.

**Method:**

A total of 160 participants (81 H. pylori-positive and 79 H. pylori-negative patients) were enrolled in this study. All participants underwent standardized neuropsychological assessments and venous blood collection. Systemic inflammation indices were derived from routine hematological parameters.

**Results:**

(1) H. pylori-positive patients showed significantly higher anxiety scores [Hamilton Anxiety Scale (HAMA) and Self-Rating Anxiety Scale (SAS)] and elevated inflammatory markers [systemic immune-inflammation index (SII), systemic inflammation response index (SIRI), and aggregate index of systemic inflammation (AISI)] as compared to H. pylori-negative ones. (2) AISI showed optimal diagnostic accuracy for infection status [area under the curve (AUC) =0.746)], followed by SIRI and SII (both AUC > 0.7). (3) In H. pylori-positive patients, inflammatory markers correlated with both anxiety scores and glial fibrillary acidic protein (GFAP) levels. (4) Interactions between serum GFAP and blood SII and AISI were significantly associated with HAMA and SAS scores in H. pylori-positive patients. (5) The GFAP as the mediator, affected the relationship between the blood levels of systemic inflammatory markers and HAMA and SAS scores in H. pylori-positive patients.

**Conclusion:**

Our findings suggest systemic inflammation indices contribute to anxiety development in H. pylori infection and may serve as practical biomarkers for anxiety screening.

## Introduction

Helicobacter pylori (H. pylori), a Gram-negative, microaerobic bacterium of the Proteobacteria phylum, colonizes the stomach and duodenum, contributing to gastrointestinal disorders, including gastritis, peptic ulcers, and gastric cancer (1, 2). Chronic H. pylori infection can exert systemic effects via gut-brain axis modulation and induce neuroinflammation, potentially reaching the central nervous system (CNS) through hematogenous spread, olfactory pathways, or retrograde axonal transport from the gut (3, 4). Growing evidence suggests that H. pylori infection may contribute to neurological disorders, including Alzheimer’s disease, stroke, multiple sclerosis (5). Furthermore, H. pylori infection has been strongly associated with psychiatric conditions, particularly anxiety (6–9). Notably, even after successful eradication therapy, some individuals may develop anxiety disorders due to persistent concerns about infection recurrence or fear of severe illness, despite testing negative for H. pylori antigens. A previous clinical study indicates that approximately 30% of H. pylori-positive individuals developed anxiety-related complications and psychiatric intervention can obviously enhance treatment outcomes for H. pylori infection (10). Therefore, early detection of anxiety in H. pylori-infected patients is clinically important, and the identification of reliable biomarkers for anxiety could significantly improve therapeutic management of H. pylori infection.

Due to the systemic inflammatory response serves as the key link between H. pylori infection and anxiety, inflammatory-related markers show more potential. Recent advances in inflammatory assessment have utilized leukocyte subtype ratios from complete blood counts to quantify systemic inflammation (11). Three novel indices have emerged as particularly valuable: the Systemic Immune-Inflammation Index (SII), Systemic Inflammation Response Index (SIRI), and Aggregate Index of Systemic Inflammation (AISI), which have demonstrated significant diagnostic and prognostic utility across various clinical conditions (12, 13). Previous study has demonstrated that elevated SIRI levels are significantly associated with higher all-cause mortality in H. pylori-infected individuals (14). Meanwhile, these hematological inflammatory markers show potential as screening biomarkers for early-stage gastric cancer detection (15, 16). Furthermore, increased levels of these systemic inflammatory markers have also been observed in patients with anxiety disorders, with blood concentrations positively correlating with anxiety severity (17, 18). The clinical applicability of these systemic inflammatory markers for H. pylori-positive individuals with comorbid anxiety has not yet been established, warranting investigation into their potential effects on central nervous system neuroinflammation.

Astrocyte activation can induce neuroinflammation, subsequently affecting brain regions implicated in mood regulation, including anxiety-related circuits (19). Serum glial fibrillary acidic protein (GFAP), a peripheral biomarker of astrocyte activation, has been shown to both reflect the degree of astrocyte-mediated neuroinflammation and correlate with the pathogenesis of anxiety disorders (20–22). In our previous study, serum GFAP levels were found to differ significantly between H. pylori-positive and H. pylori-negative patients (23). Furthermore, among H. pylori-positive individuals, GFAP levels showed a significant correlation with scores on depressive psychological assessments (23).

This study aims to: (1) compare the expression levels of systemic inflammatory markers between H. pylori-positive and H. pylori-negative patients; (2) assess the association between these markers and anxiety symptoms in H. pylori-positive individuals; and (3) investigate the potential mechanisms through which these inflammatory markers may influence anxiety pathology in H. pylori-infected patients. The hypothesis of this study was that systemic inflammatory marker levels differed significantly between the two groups and were correlated with the severity of anxiety in H. pylori-positive individuals.

## Method and materials

### Participants

A total of 160 adult Chinese Han participants were recruited from Wuxi Xishan People’s Hospital, comprising 79 H. pylori-positive patients and 81 H. pylori-negative patients. In the present study, H. pylori infection status was assessed using the^14^C-urea breath test, rapid urease test, and esophagogastroduodenoscopy (EGD) with histopathological examination of biopsy samples (**Supplementary Figure 1**). Each participant underwent all three tests. H. pylori infection was defined as a positive result in ≥ 1 test, while H. pylori-negative status required no positive findings in any of the tests. A qualified gastroenterologist conducted standardized clinical interviews and evaluated the Gastrointestinal Symptom Rating Scale (GSRS) for all participants.

Eligible participants were required to meet the following criteria: (1) age between 20 and 85 years; (2) self-reported gastrointestinal discomfort; and (3) willingness to undergo upper endoscopy and biopsy for diagnostic evaluation. Meanwhile, participants were excluded if they met any of the following conditions: (1) recent use (within one month prior to enrollment) of medications that could influence gastrointestinal function, including histamine-2 receptor antagonists, proton pump inhibitors, antibiotics, non-steroidal anti-inflammatory drugs, or probiotics; (2) a history of severe systemic diseases, such as hepatic, cardiovascular, renal, or thyroid dysfunction, as well as malignancy, active infections, stroke, or autoimmune disorders; (3) diagnosed psychiatric conditions (e.g., bipolar disorder, schizophrenia, dementia) or a family history of psychotic disorders; (4) prior surgery involving the digestive tract; (5) current alcohol or substance abuse or dependence; (6) pregnancy or lactation at the time of screening; or (7) taking psychotropic medications, including anti-anxiety or antidepressant drugs.

The present study was approved by the Ethics Committee of the Wuxi Xishan People’s Hospital (approval number: xs2021ky025). All participants or their legal guardians provided written informed consent.

### Neuropsychological assessments

Trained researchers conducted standardized neuropsychological assessments for all participants. Anxiety symptoms were evaluated using two validated scales: the Hamilton Anxiety Scale (HAMA) (24), administered by the assessors, and the Self-Rating Anxiety Scale (SAS) (25), a self-reported measure.

### Measurement of blood indices

Fasting peripheral venous blood samples were collected from each participant between 8:00 am and 9:00 am using two types of vacutainer tubes: a plain tube (without anticoagulant) and an EDTA-coated tube.

For serum preparation, blood samples in plain tubes were centrifuged at 3500 rpm for 10 minutes at 4 °C within 30 minutes of collection. The obtained serum was aliquoted and stored at -80 °C until analysis. Serum concentrations of GFAP were quantified using commercially available enzyme-linked immunosorbent assays kits (FineTest, Wuhan, China; Catalog Numbers: EH0410). All measurements were performed in triplicate, with both inter- and intra-assay coefficients of variation < 5%.

Blood samples collected in EDTA-coated tubes were immediately transported to the clinical laboratory of Wuxi Xishan People’s Hospital for complete blood count analysis. Based on these results, we calculated three systemic inflammation indices: (1) SII = (neutrophil count × platelet count)/lymphocyte count; (2) SIRI = (neutrophil count × monocyte count)/lymphocyte count; and (3) AISI = (neutrophil count × monocyte count × platelet count)/lymphocyte count, which is also referred to as systemic immune-inflammation response index.

### Statistical analyses

All statistical analyses were performed using SPSS software (version 20.0; SPSS Inc., Chicago, IL, USA). Data normality was assessed using the Kolmogorov-Smirnov test. Continuous variables with normal distribution were expressed as mean ± standard deviation and compared using independent samples t-tests, while non-normally distributed variables were analyzed using the Mann-Whitney U test. Categorical variables were compared using chi-square tests. The diagnostic performance of biomarkers was evaluated through receiver operating characteristic (ROC) curve analysis, with the area under the curve (AUC) serving as the primary accuracy measure. Optimal cutoff values were determined by maximizing the Youden index (26, 27).

Bivariate correlations were examined to assess relationships between continuous variables. Linear regression analysis was employed to evaluate multivariate associations with anxiety symptoms (28). Additionally, we conducted mediation analyses to investigate whether serum markers mediated the relationship between systemic inflammation indices and anxiety symptom severity, following established three-variable mediation models (29–31). A two-tailed p-value < 0.05 was considered statistically significant for all analyses.

## Results

### Comparative analysis of clinical characteristics between *H. pylori*-positive and negative groups

As shown in **Table 1**, the two groups demonstrated comparable baseline characteristics including age, sex, endoscopic findings, and GSRS scores (all p > 0.05). However, H. pylori-positive patients exhibited significantly higher anxiety scores on both the HAMA and SAS compared to the H. pylori-negative ones (p < 0.05). Meanwhile, serum analysis demonstrated significant between-group differences in GFAP levels (p < 0.05).

**Table 1:** Comparison of clinical features, psychological assessments and blood indices levels between the *H. pylori*-positive and *H. pylori*-negative groups.

	H. pylori-negative (n = 81)	H. pylori-positive (n = 79)	P-value
Age (years)	56.99 ± 12.80	57.71 ± 13.22	0.726^*^
Sex (Female, %)	28 (34.57%)	30 (37.97%)	0.201^#^
Endoscopic features			0.896^#^
Superficial gastritis, %	47 (58.02%)	48 (60.76%)	
Atrophic gastritis, %	11 (13.58%)	7 (8.86%)	
erosive gastritis, %	23 (28.40%)	24 (30.38%)	
GSRS scores	8.94 ± 2.89	9.43 ± 3.07	0.276^†^
HAMA scores	4.60 ± 3.19	26.41 ± 7.02	< 0.001^†^
SAS scores	33.84 ± 5.68	63.10 ± 7.04	< 0.001^†^
Blood indices levels			
Neutrophil count, 10^9^/L	3.34 ± 1.08	4.10 ± 1.21	0.001^*^
Lymphocyte count, 10^9^/L	1.94 ± 0.64	1.94 ± 0.81	0.493^†^
Monocyte count, 10^9^/L	0.41 ± 0.13	0.49 ± 0.19	0.010^*^
Platelet count, 10^9^/L	200.62 ± 54.10	222.32 ± 55.97	0.014^*^
Erythrocyte count, 10^9^/L	4.56 ± 0.63	4.49 ± 0.76	0.495^*^
Hemoglobin, g/L	139.77 ± 18.67	134.72 ± 20.54	0.106^*^
GFAP, ng/ml	0.30 ± 0.73	0.38 ± 0.39	< 0.001^†^

Data are presented as means ± standard deviation or number of participants in each group (% of total).

H. pylori, Helicobacter pylori; GSRS, Gastrointestinal Symptom Rating Scale; HAMA, Hamilton Anxiety Scale; SAS, Self-Rating Anxiety Scale; GFAP, glial fibrillary acidic protein.

^*^P values were obtained by Independent-Samples T test.

^†^P values were obtained by Mann-Whitney U test.

^#^P values were obtained by Chi-square test.

**Figure 1:**
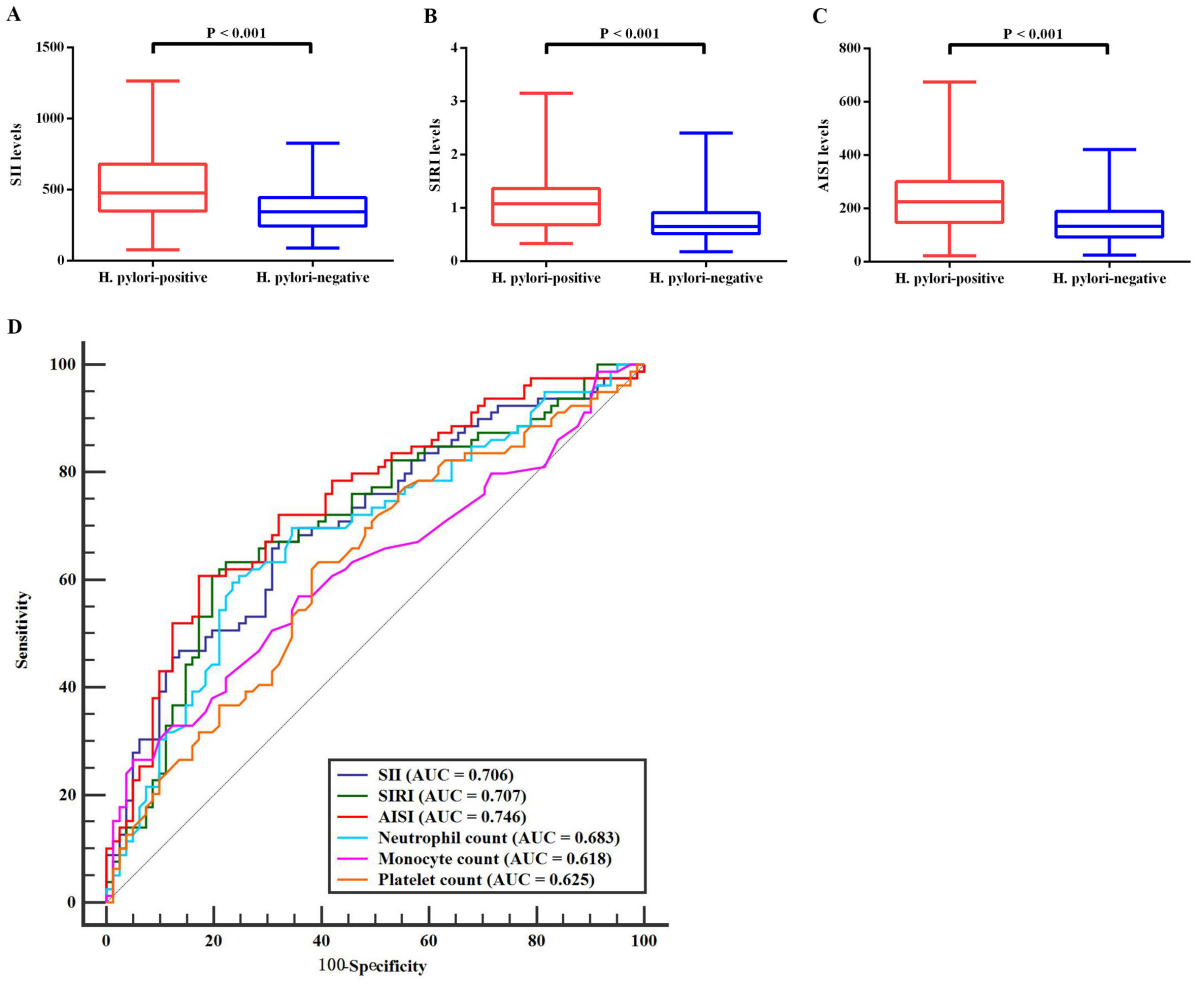
Comparative analysis of inflammation markers between *H. pylori*-positive and *H. pylori*-negative groups. **(A–C)** Significant differences in blood levels of three systemic inflammatory markers between H. pylori-infected and uninfected individuals. **(D)** ROC curve analyses for the diagnostic performance of six differentially expressed inflammation markers that showed significant variation between the two groups. H. pylori, Helicobacter pylori; SII, systemic immune-inflammation index; SIRI, systemic inflammation response index; AISI, aggregate index of systemic inflammation; ROC, receiver operating characteristic; AUC, area under the curve.

Blood count analysis indicated no significant intergroup differences in lymphocyte counts, erythrocyte counts, or hemoglobin levels (p > 0.05 for all; **Table 1**). However, H. pylori-positive patients showed distinct alterations in several inflammatory markers, including elevated neutrophil counts, monocyte counts, and platelet counts (p < 0.05; **Table 1**). Additionally, H. pylori-positive patients demonstrated significantly elevated levels of all three systemic inflammation indices (SII, SIRI, and AISI) compared to H. pylori-negative individuals (**Figures 1A–C**).

### ROC curve analysis

ROC curve analysis was conducted on the six blood count indices that showed significant differences between H. pylori-positive and negative groups (**Figure 1D**). The results revealed that the AISI demonstrated the highest diagnostic accuracy (AUC = 0.746; sensitivity = 60.76%, specificity = 82.72%), followed by the SIRI (AUC = 0.707; sensitivity = 63.29%, specificity = 77.78%) and SII (AUC = 0.706; sensitivity = 67.09%, specificity = 67.90%). The remaining three indices showed limited discriminative ability, with AUC values below 0.7.

### Psychological assessments association with systemic inflammation indices and serum indices levels in *H. pylori*-positive patients

In H. pylori-positive patients, significant positive correlations were observed between three systemic inflammation indices and serum GFAP levels (**Table 2**). Moreover, HAMA scores showed significant positive correlations with all three systemic inflammation indices in these patients, while SAS scores were only positively associated with SII and AISI levels (**Table 2**).

**Table 2:** Correlation analyses between blood systemic inflammatory markers levels and serum GFAP levels and psychological assessments in the *H. pylori*-positive group.

	SII levels	SIRI levels	AISI levels
GFAP levels	r = 0.270, p = 0.016	r = 0.273, p = 0.015	r = 0.380, p = 0.001
HAMA scores	r = 0.383, p < 0.001	r = 0.289, p = 0.010	r = 0.353, p = 0.001
SAS scores	r = 0.370, p = 0.001	r = 0.181, p = 0.110	r = 0.263, p = 0.019

H. pylori, Helicobacter pylori; SII, systemic immune-inflammation index; SIRI, systemic inflammation response index; AISI, aggregate index of systemic inflammation; GFAP, glial fibrillary acidic protein; HAMA, Hamilton Anxiety Scale; SAS, Self-Rating Anxiety Scale.

**Figure 2:**
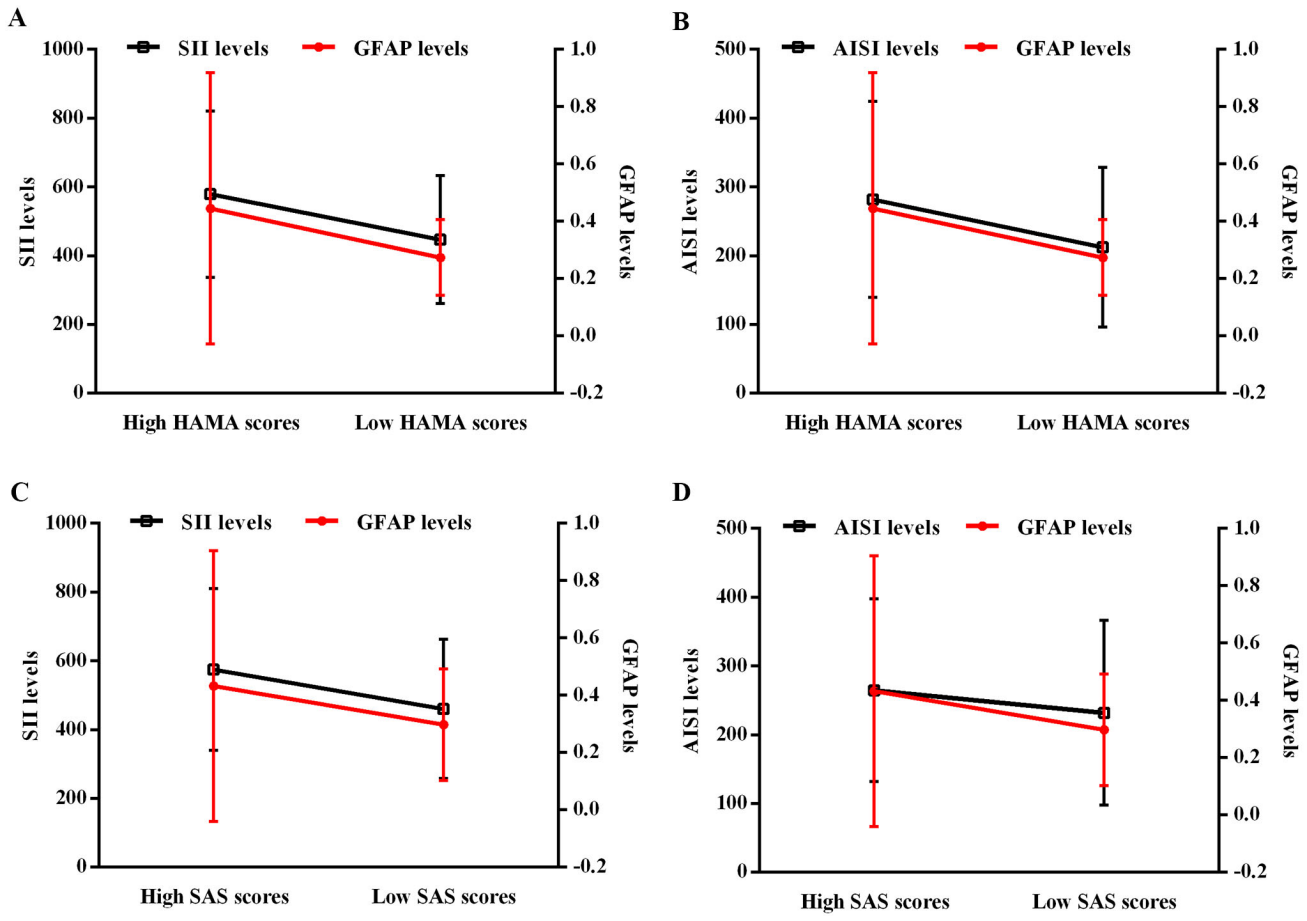
Regression analyses of the interactive effect between blood SII/AISI and serum GFAP on HAMA/SAS score in *H. pylori*-positive patients. **(A)** SII and GFAP for HAMA. **(B)** AISI and GFAP for HAMA. **(C)** SII and GFAP for SAS. **(D)** AISI and GFAP for SAS. H. pylori, Helicobacter pylori; SII, systemic immune-inflammation index; AISI, aggregate index of systemic inflammation; GFAP, glial fibrillary acidic protein; HAMA, Hamilton Anxiety Scale; SAS, Self-Rating Anxiety Scale.

In addition, linear regression analyses revealed that blood SII, AISI, and serum GFAP levels were significantly associated with anxiety in H. pylori-positive patients (**Figure 2**). To further characterize these relationships, we stratified H. pylori-positive patients into two subgroups based on their HAMA scores: a “low HAMA” group (n = 39, HAMA score < 27) and a “high HAMA” group (n = 40, HAMA score ≥ 27), using the mean HAMA score as the cutoff. The results demonstrated that patients in the high HAMA group exhibited significantly elevated blood SII and AISI levels, along with higher serum GFAP levels, compared to the low HAMA group (**Figures 2A, B**). Likewise, we classified H. pylori-positive patients into “low SAS” (n = 42, SAS score < 64) and “high SAS” (n = 37, SAS score ≥ 64) subgroups based on the mean SAS score. Consistent with the HAMA findings, patients with higher SAS scores also displayed increased SII, AISI, and GFAP levels (**Figures 2C, D**).

**Figure 3:**
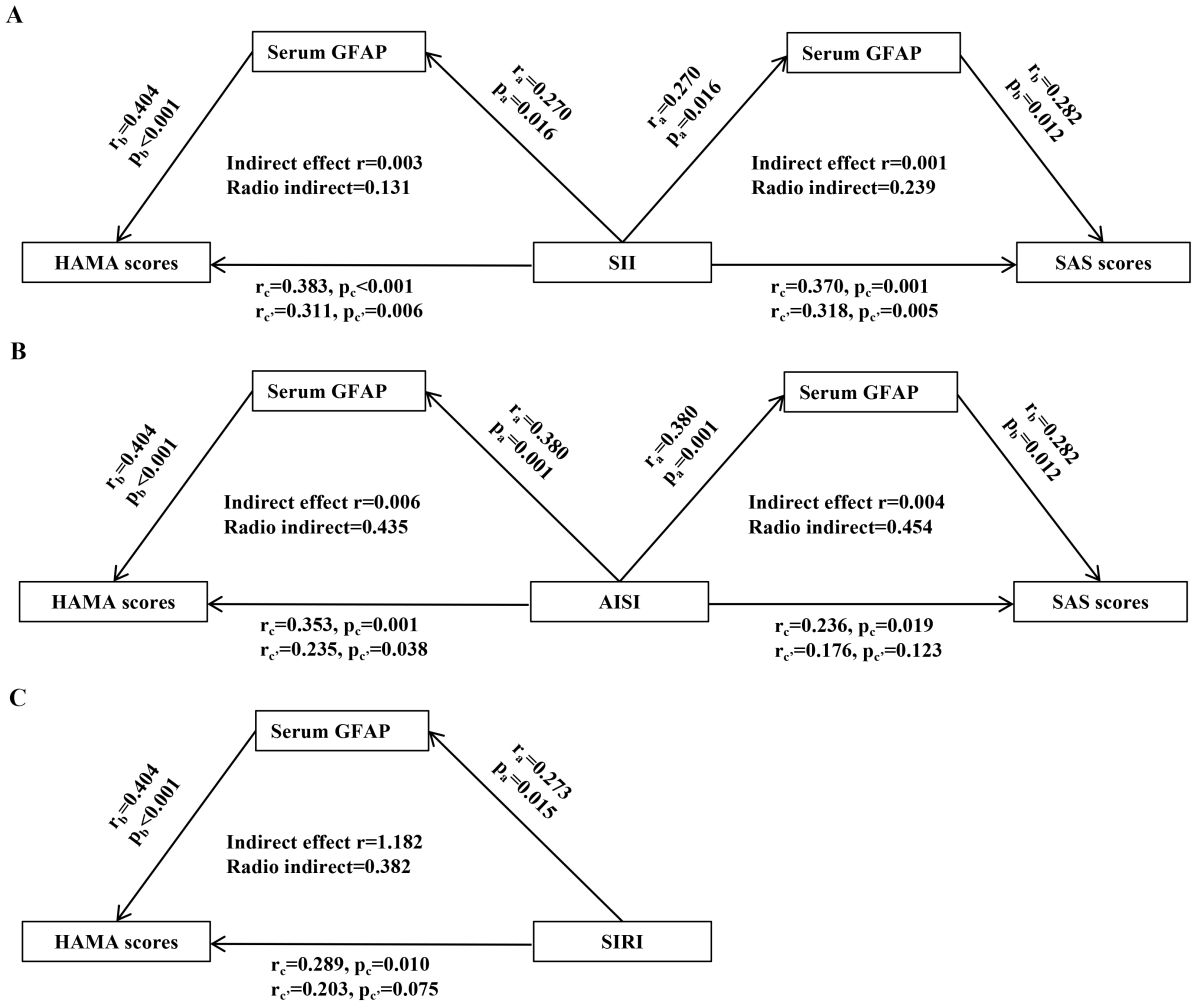
Mediating role of serum GFAP in the relationship between systemic inflammation and anxiety scores in *H. pylori*-positive patients. **(A)** SII. **(B)** AISI. **(C)** SIRI. The results of mediation analysis reveal that serum GFAP was able to modulate the association between systemic inflammatory markers levels and HAMA and SAS scores. “a,” “b,” and “c′” represent the direct effect between the 2 variables, and “c” represents the total effect between the 2 variables. H. pylori, Helicobacter pylori; SII, systemic immune-inflammation index; SIRI, systemic inflammation response index; AISI, aggregate index of systemic inflammation; GFAP, glial fibrillary acidic protein; HAMA, Hamilton Anxiety Scale; SAS, Self-Rating Anxiety Scale.

Further mediation analyses in H. pylori-positive patients demonstrated that serum GFAP significantly mediated the relationship between systemic inflammation indices and anxiety severity (**Figure 3**). Specifically, serum GFAP levels indirectly influenced the association of SII with both HAMA and SAS scores, as well as the association of AISI with HAMA scores (**Figures 3A, B**). Notably, GFAP also exhibited a direct mediating effect on the relationship between AISI and SAS scores, as well as between SIRI and HAMA scores (**Figures 3B, C**).

## Discussion

In the present study, we mainly found that (1) H. pylori-positive patients exhibited significantly elevated HAMA and SAS scores, along with increased blood levels of SII, SIRI, and AISI compared to H. pylori-negative controls; (2) among the inflammatory indices, AISI demonstrated superior discriminative power for identifying H. pylori infection, followed by SIRI and SII; (3) significant correlations were observed between systemic inflammatory markers (SII/SIRI/AISI) and both psychological assessment scores and serum GFAP levels in H. pylori-positive patients; (4) interaction analyses revealed that GFAP significantly moderated the effects of SII and AISI on anxiety severity (HAMA/SAS scores); and (5) GFAP-mediated neuroinflammation may underlie the association between systemic inflammation and anxiety severity. Altogether, these findings indicate that systemic inflammation indices may contribute to anxiety pathogenesis in H. pylori infection and could serve as clinically useful biomarkers for anxiety detection in this patient population.

Consistent with previous findings (10, 32), our study demonstrated that H. pylori-positive patients exhibited significantly higher scores on both the HAMA and the SAS compared to H. pylori-negative individuals, suggesting a potential association between H. pylori infection and the manifestation of anxiety symptoms. H. pylori’s persistent colonization in the host’s stomach can trigger and sustain systemic inflammation, which may contribute to neuroinflammatory processes (23, 33, 34). Previous studies have demonstrated that elevated levels of pro-inflammatory cytokines and immune system activation can exacerbate anxiety-like behaviors (35, 36), suggesting a link between inflammation and anxiety. Based on these evidence, we focused on systemic inflammatory markers to evaluate the potential association between H. pylori infection and anxiety. In the present study, blood levels of three systemic inflammatory markers (SII, SIRI, and AISI) were significantly higher in H. pylori-positive patients than in H. pylori-negative individuals, supporting that H. pylori infection is associated with severe systemic inflammation. Subsequently, the correlation analyses further revealed that blood levels of SII, SIRI, and AISI were positively associated with anxiety severity (as assessed by HAMA and SAS scores) in H. pylori-positive patients, suggesting that these systemic inflammatory markers may contribute to the development of anxiety symptoms following H. pylori infection. Among other digestive system disorders (e.g., inflammatory bowel disease), elevated peripheral inflammatory responses have been also identified as a key mechanism underlying the occurrence of anxiety (37). The current findings provide valuable evidence supporting the association between peripheral systemic inflammatory markers and H. pylori-related anxiety. Furthermore, ROC curve analysis demonstrated that these systemic inflammatory markers exhibited good diagnostic performance in differentiating H. pylori-positive from H. pylori-negative individuals. Hence, SII, SIRI, and AISI may serve as potential biomarkers for early diagnosis of H. pylori-positive patients with anxiety, potentially facilitating early anti-anxiety intervention following H. pylori infection.

In our study, we observed that potential interactions between serum GFAP and both SII and AISI levels could influence anxiety severity in H. pylori-positive patients. This finding suggests that systemic inflammation may contribute to anxiety through astrocyte-mediated neuroinflammation. Subsequent mediation analyses further revealed that serum GFAP potentially affected the relationship between systemic inflammatory markers and anxiety severity in these patients. Therefore, GFAP-associated astrocyte activation as a critical link may connect systemic inflammation with anxiety development in patients with H. pylori infection.

Furthermore, there is a strong association between H. pylori infection and anxiety, suggesting that anxiety may also serve as a potential risk factor for H. pylori infection. Although direct evidence linking anxiety to an increased susceptibility to H. pylori is limited, anxiety has been associated with gastrointestinal disturbances and a higher risk of various gastrointestinal disorders, such as gastroesophageal reflux disease, gastric ulcers, and gastritis (38). Given that the microbiota-gut-brain axis represents a bidirectional communication system (39–41), it is plausible that gut microbiota dysbiosis resulting from anxiety could compromise gastrointestinal function, thereby potentially increasing the likelihood of acquiring H. pylori infection. Meanwhile, although less likely, anxiety may also induce neuroinflammation—such as through the release of serum GFAP—which could potentially increase systemic inflammation and gastrointestinal susceptibility to H. pylori infection. Therefore, further animal studies are needed to evaluate the causal relationship between anxiety and H. pylori infection.

The present study had several limitations. (1) Our study was based on cross-sectional data, limiting our ability to assess longitudinal changes in systemic inflammatory markers. Furthermore, it remains unclear whether the observed association between these markers and anxiety persists following H. pylori eradication therapy. (2) Further animal studies are warranted to validate these findings and elucidate the underlying mechanisms. Future investigations should examine both peripheral and CNS levels of systemic inflammatory markers and GFAP in mouse models of H. pylori infection, with particular focus on whether modulation of these factors through human intervention can alter anxiety-like behaviors. (3) The systemic inflammatory response should be further characterized through comprehensive assessment of additional inflammatory mediators, including proinflammatory cytokines and chemokines.

## Conclusion

The present study revealed significantly higher HAMA and SAS scores in H. pylori-positive patients, suggesting the presence of anxiety-like symptoms associated with H. pylori infection. These findings may be explained by circulating systemic inflammatory markers potentially triggering astrocyte activation, which could contribute to anxiety development in infected patients.

## Data Availability

The original contributions presented in the study are included in the article/**Supplementary Material**. Further inquiries can be directed to the corresponding authors.
