# Supplementary material for: Associations between blood systemic inflammatory markers and anxiety in Helicobacter pylori-infected patients
Source: Front Psychiatry. 2025 Oct 23;16:1671858. doi: 10.3389/fpsyt.2025.1671858 (PMC12589537; doi:10.3389/fpsyt.2025.1671858)
Supplement: Supplementary file 1 [file DataSheet1.docx]

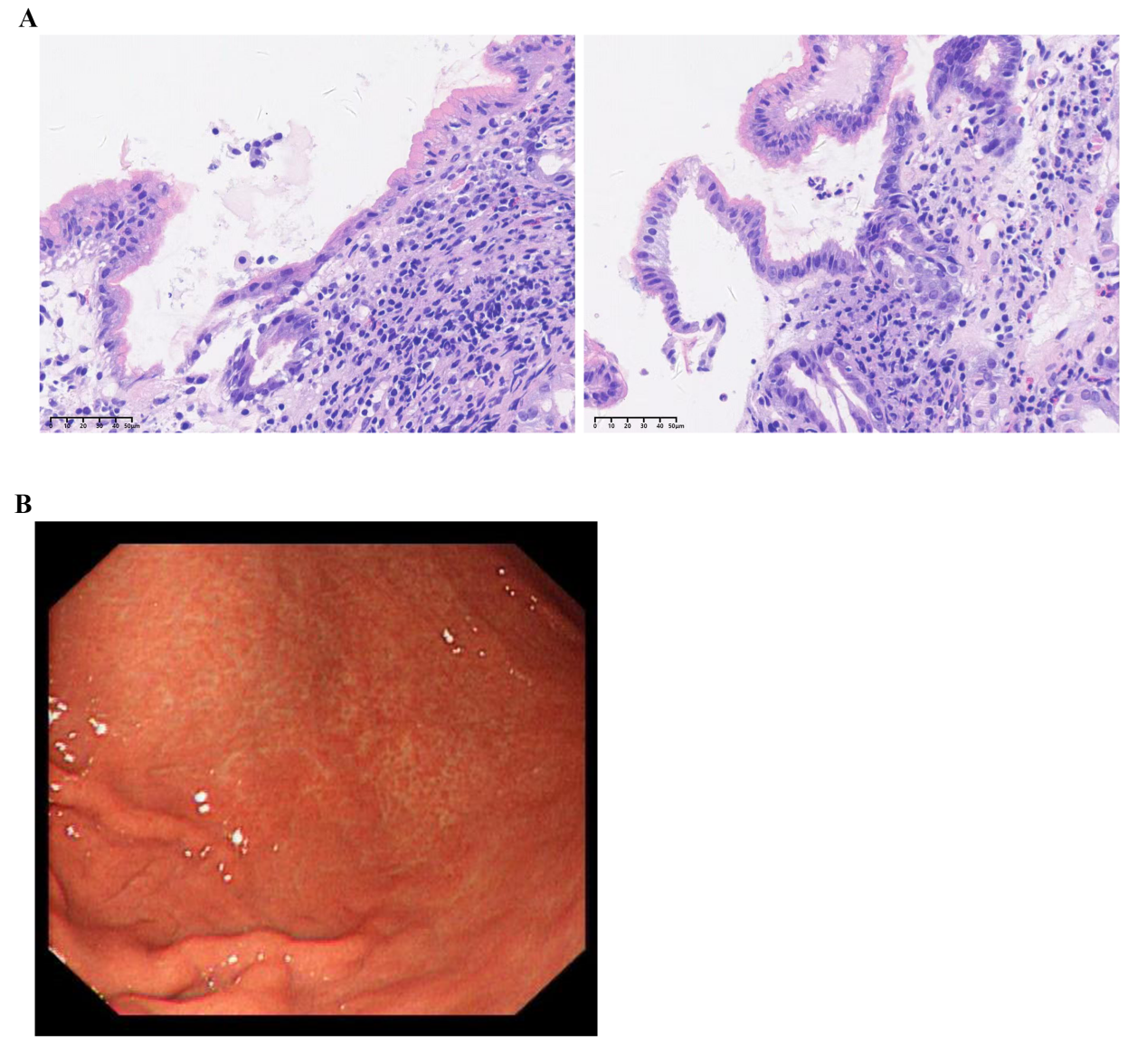


**Supplementary Figure 1. The biopsy and endoscopy analysis**

(A) The biopsy analysis of H. pylori infection. The black arrow is the H. pylori and specimens were taken from the vicinity of the gastric antrum. (B) The endoscopy analysis of the gastric antrum.
